# Supplementary material for: miRNA Mediated Noise Making of 3′UTR Mutations in Cancer
Source: Genes (Basel). 2018 Nov 12;9(11):545. doi: 10.3390/genes9110545 (PMC6267165; doi:10.3390/genes9110545)
Supplement: Supplementary file 1 [file genes-09-00545-s001.zip › genes-382433-Supplementary/genes-382433-Supplementary Figures.docx]

**
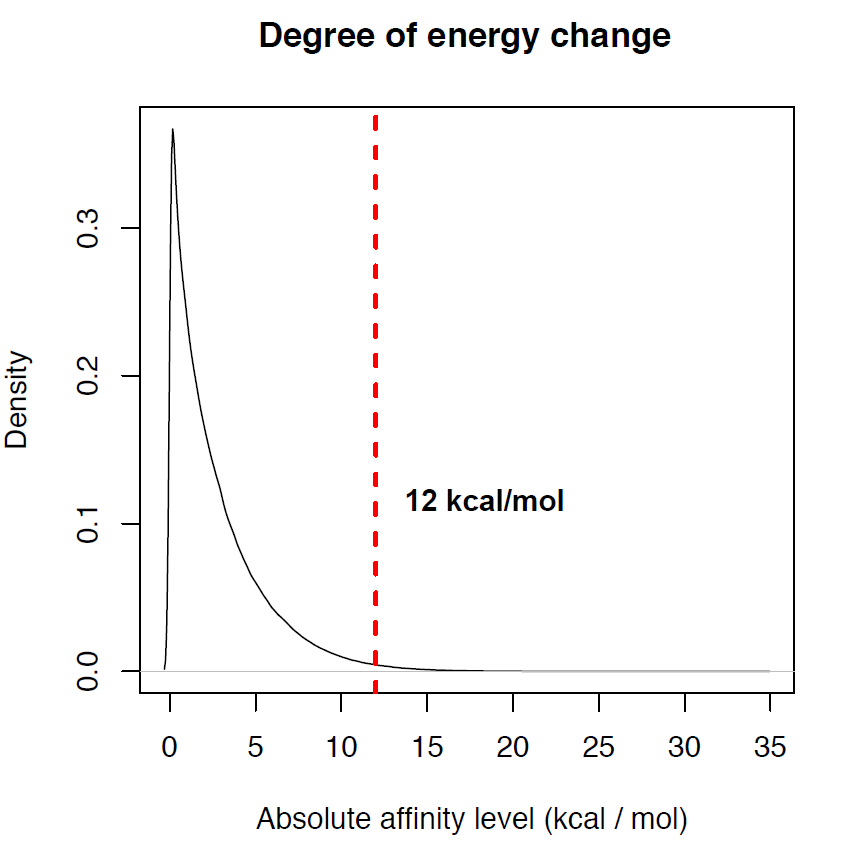
Figure S1.** The distribution of absolute affinity.


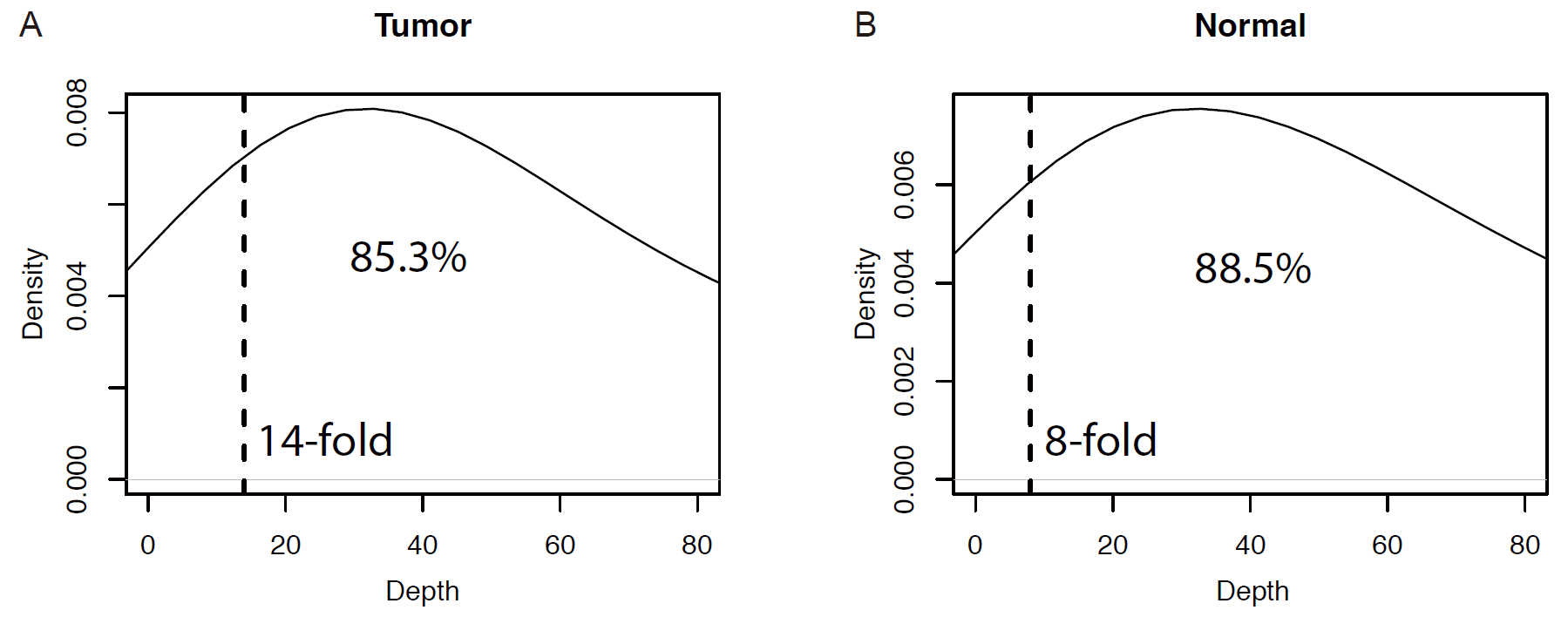


**Figure S2.** The density of depth in 3’UTR.

**
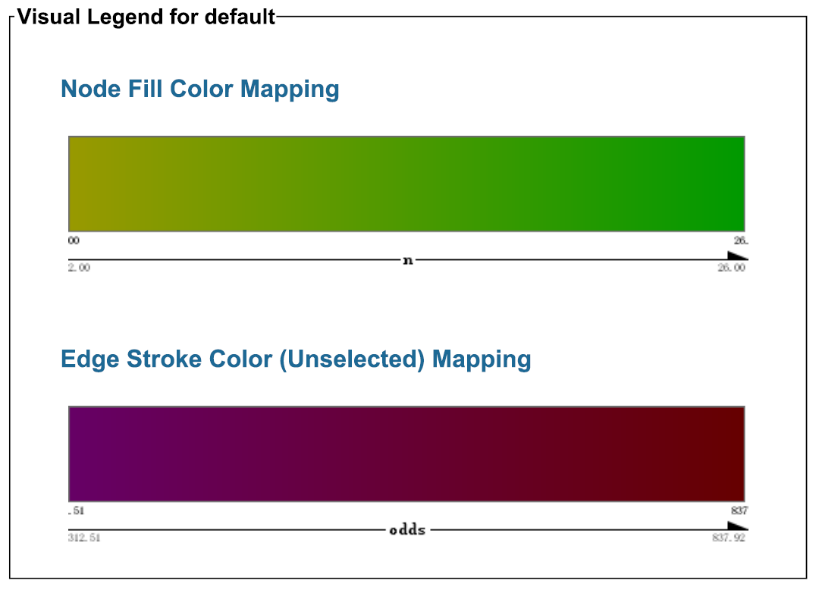
**


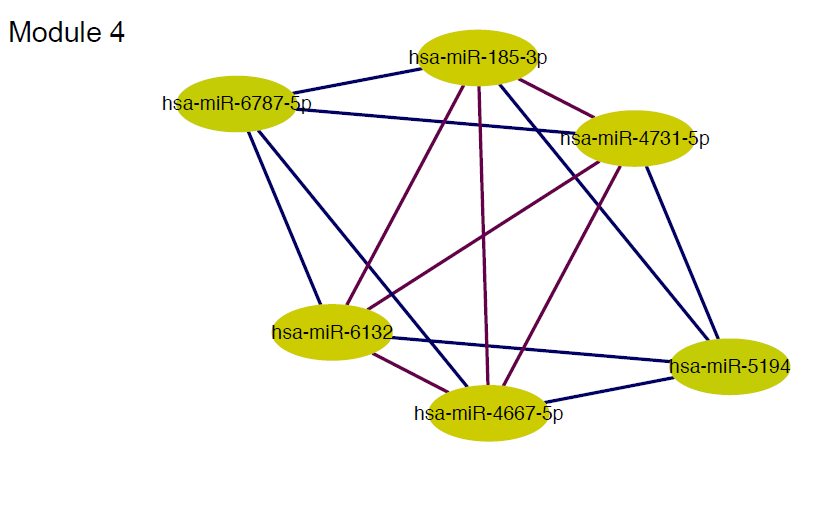

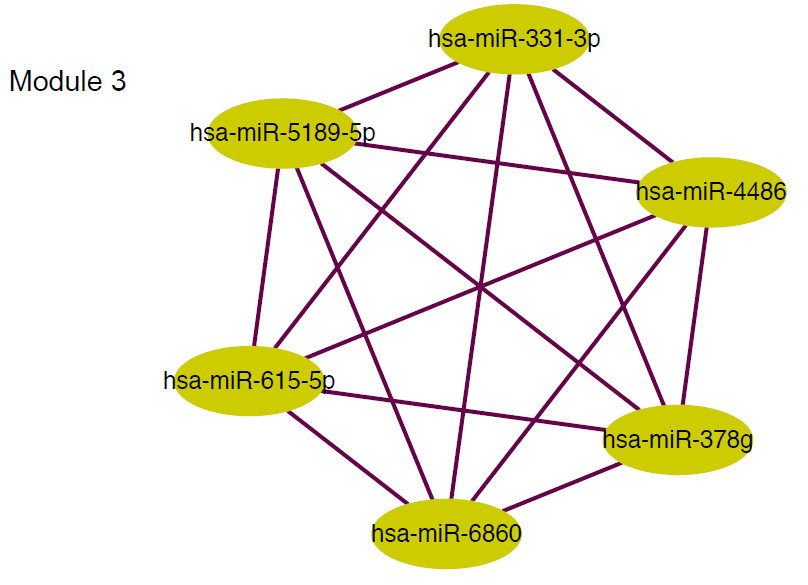


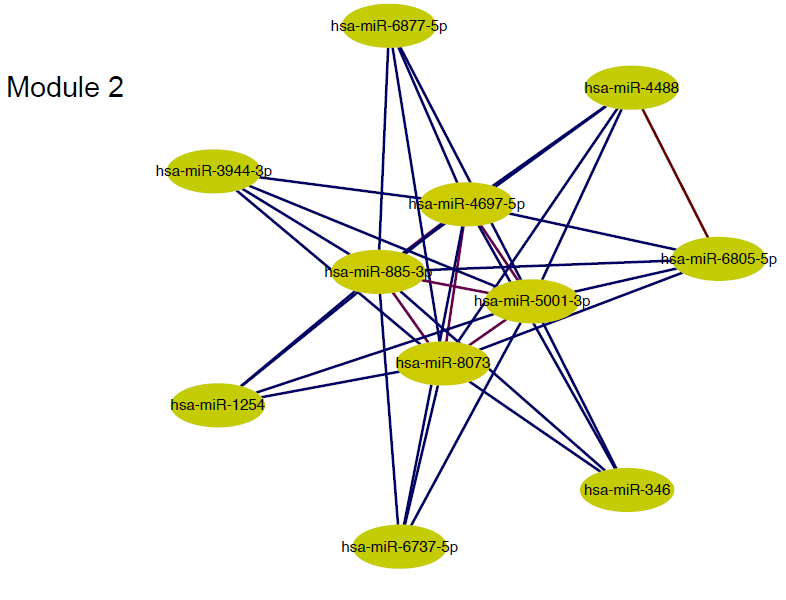

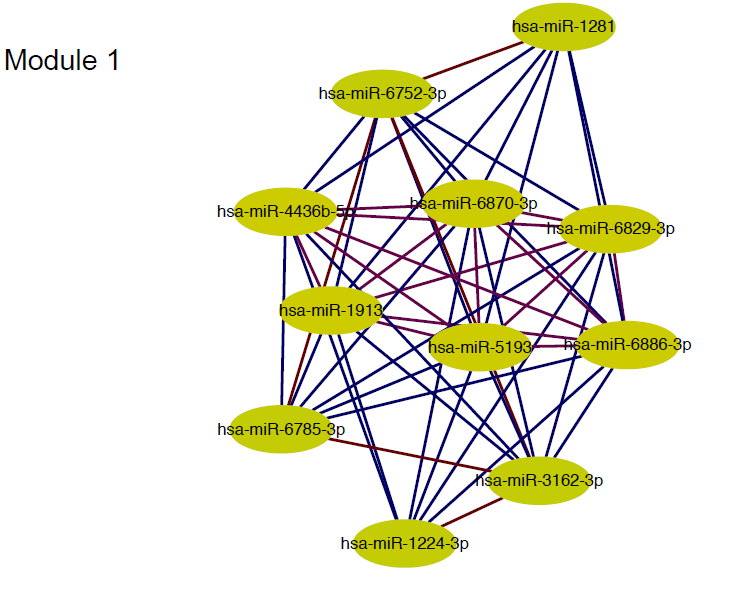


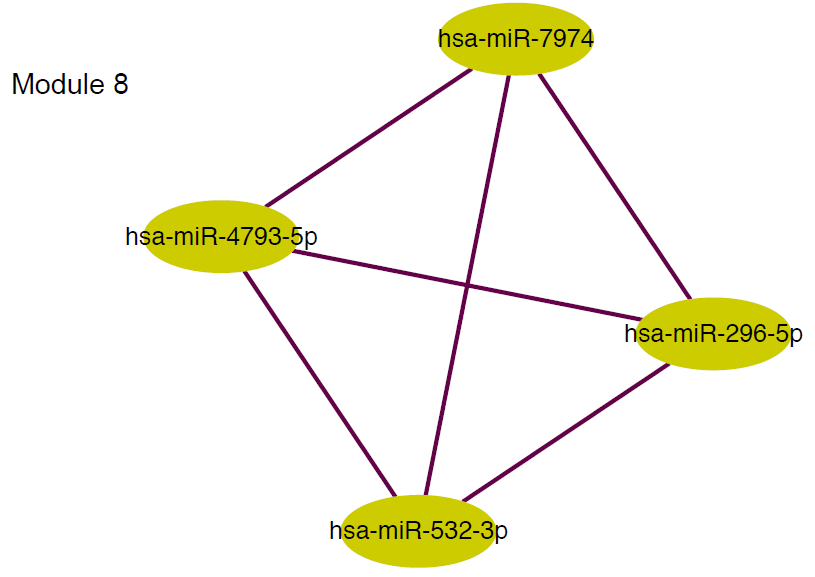

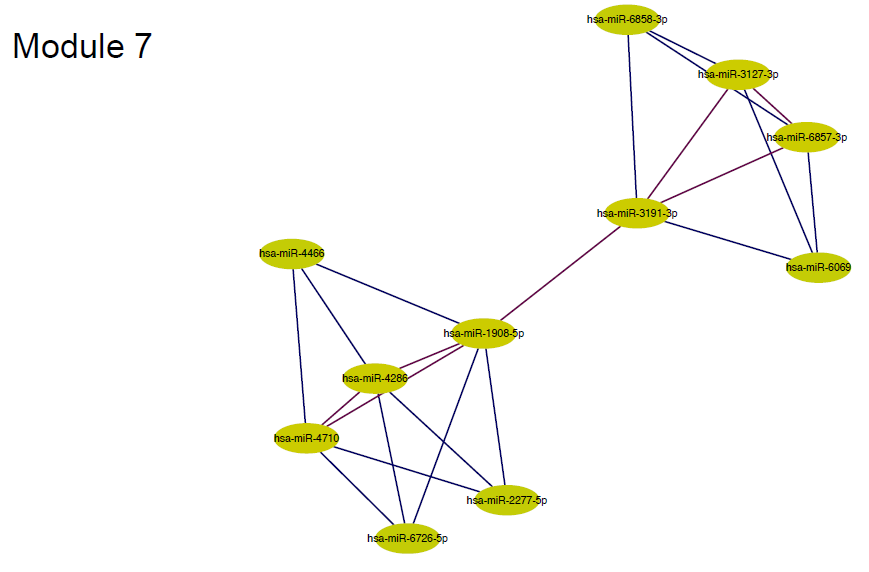

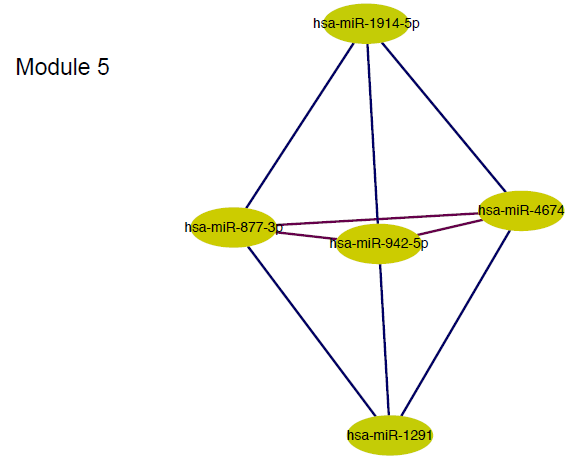

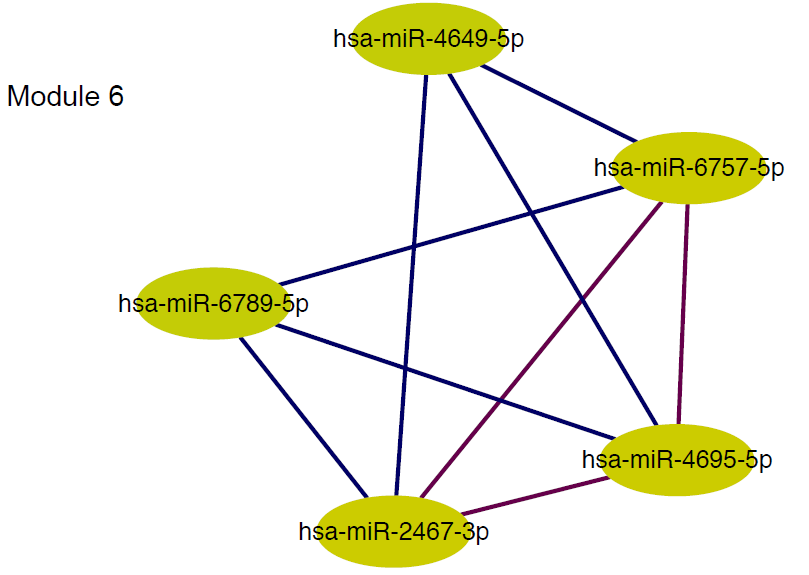


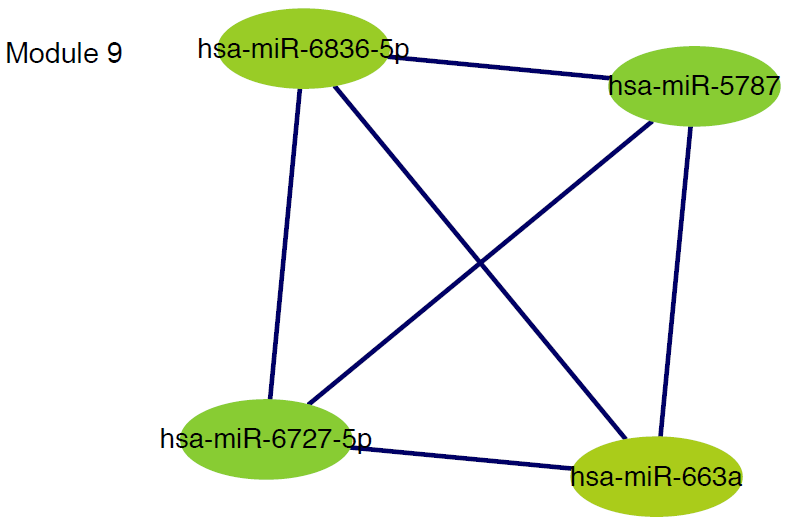


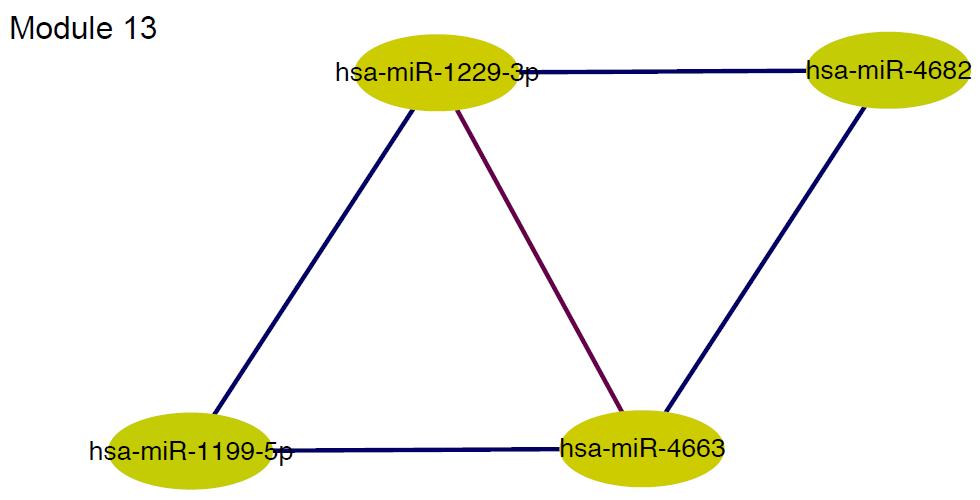

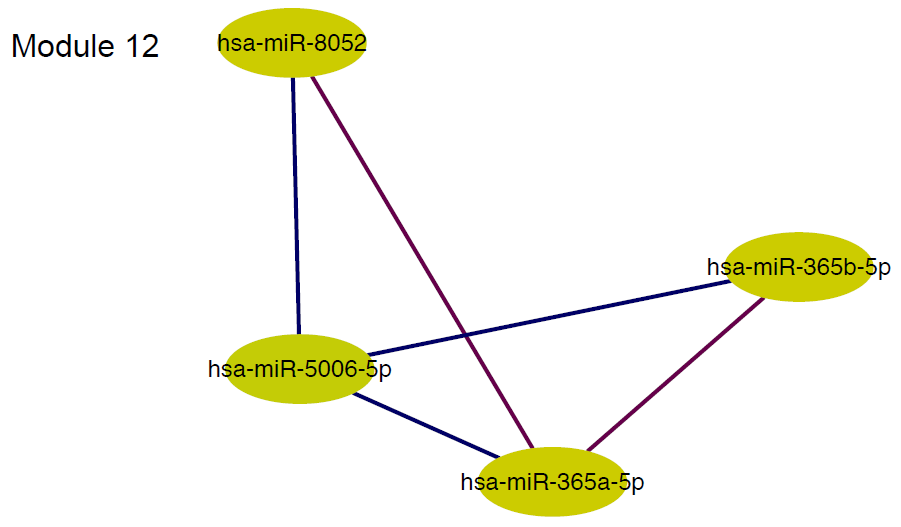

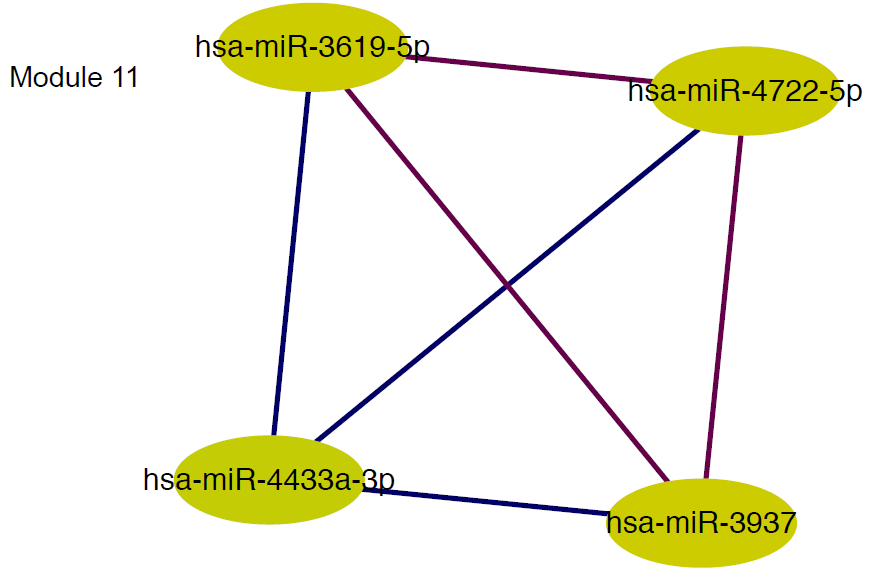

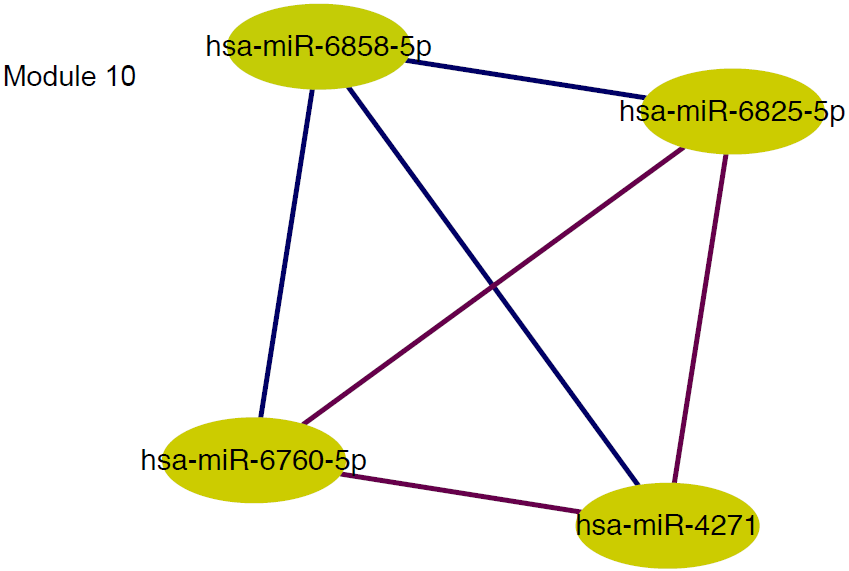


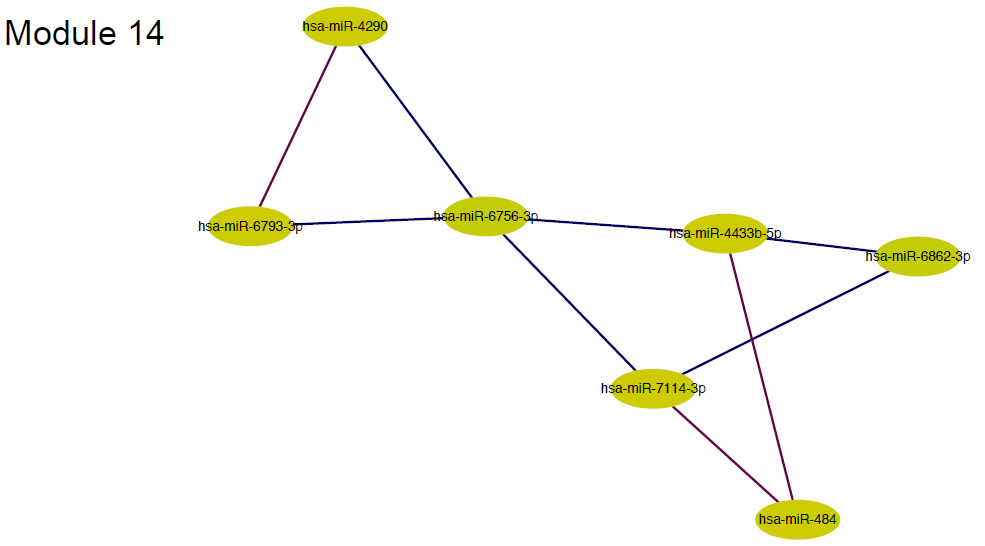


**
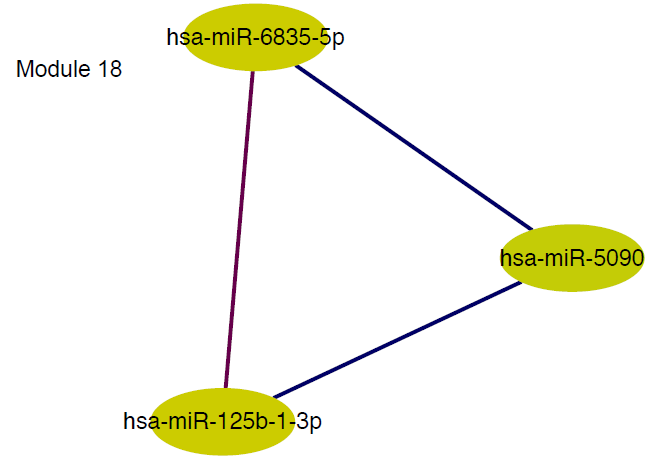

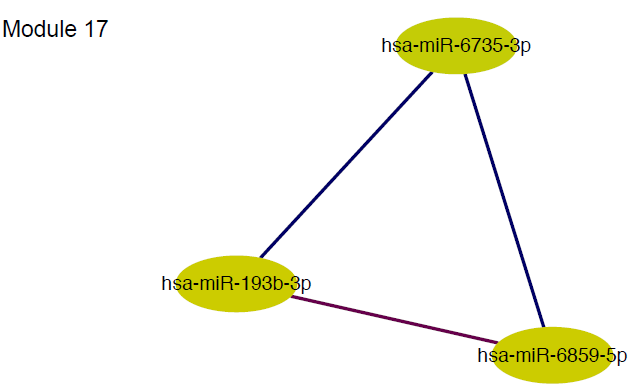

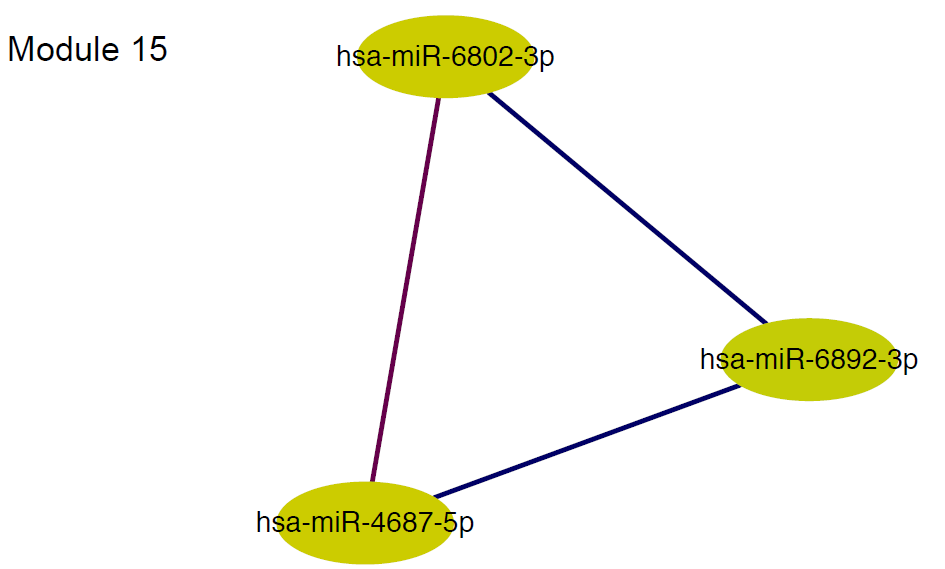

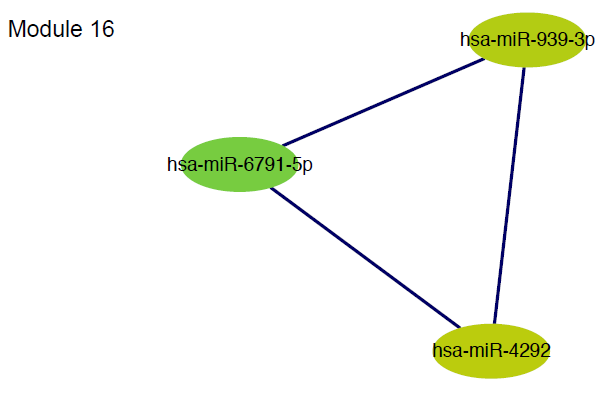
**

**Figure S3.** Eighteen modules of miRNAs in gain status.

© 2018 by the authors. Submitted for possible open access publication under the terms and conditions of the Creative Commons Attribution (CC BY) license (http://creativecommons.org/licenses/by/4.0/).
