# Supplementary material for: miRNA Mediated Noise Making of 3′UTR Mutations in Cancer
Source: Genes (Basel). 2018 Nov 12;9(11):545. doi: 10.3390/genes9110545 (PMC6267165; doi:10.3390/genes9110545)
Supplement: Supplementary file 1 [file genes-09-00545-s001.zip › genes-382433-Supplementary/Supplement Figure S3.pdf]

Visual Legend for default

Node Fill Color Mapping

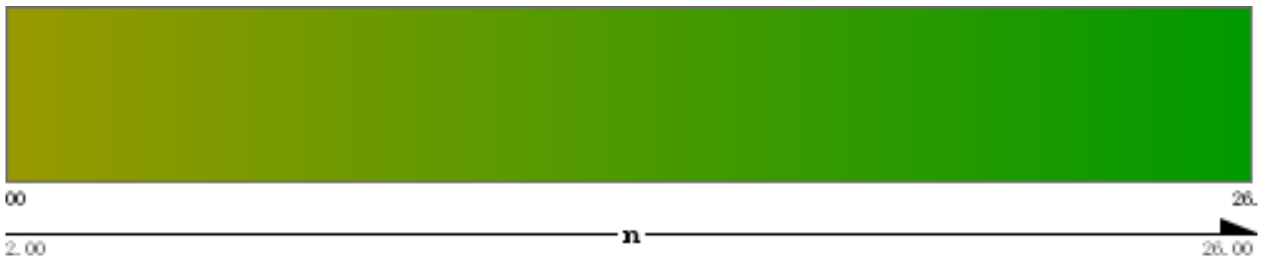

Edge Stroke Color (Unselected) Mapping

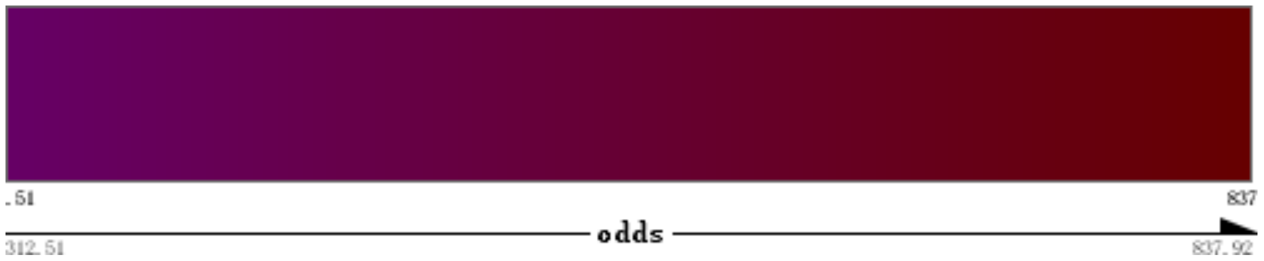

Module 1

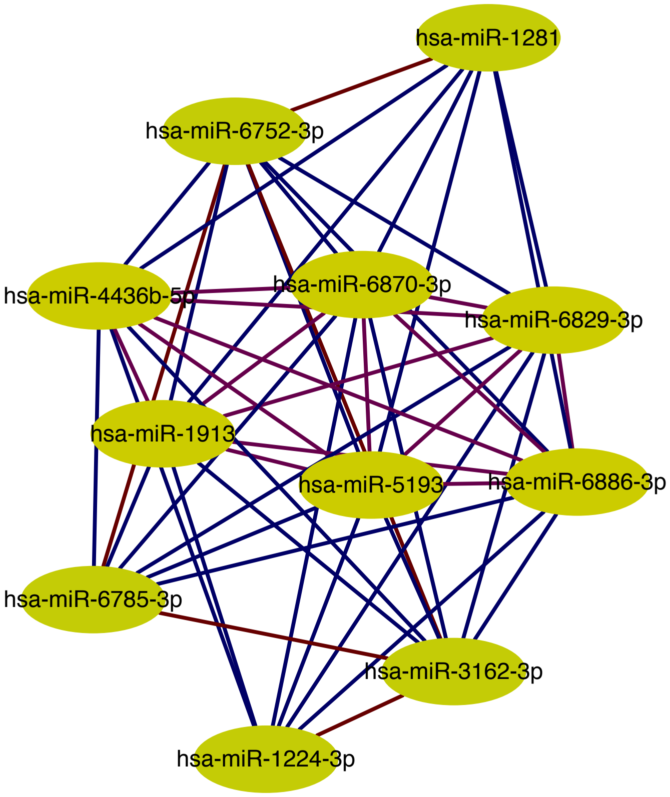
hsa-miR-1281
hsa-miR-6752-3p
hsa-miR-4436b-5p
hsa-miR-6870-3p
hsa-miR-6829-3p
hsa-miR-1913
hsa-miR-5193
hsa-miR-6886-3p
hsa-miR-6785-3p
hsa-miR-3162-3p
hsa-miR-1224-3p

Module 2

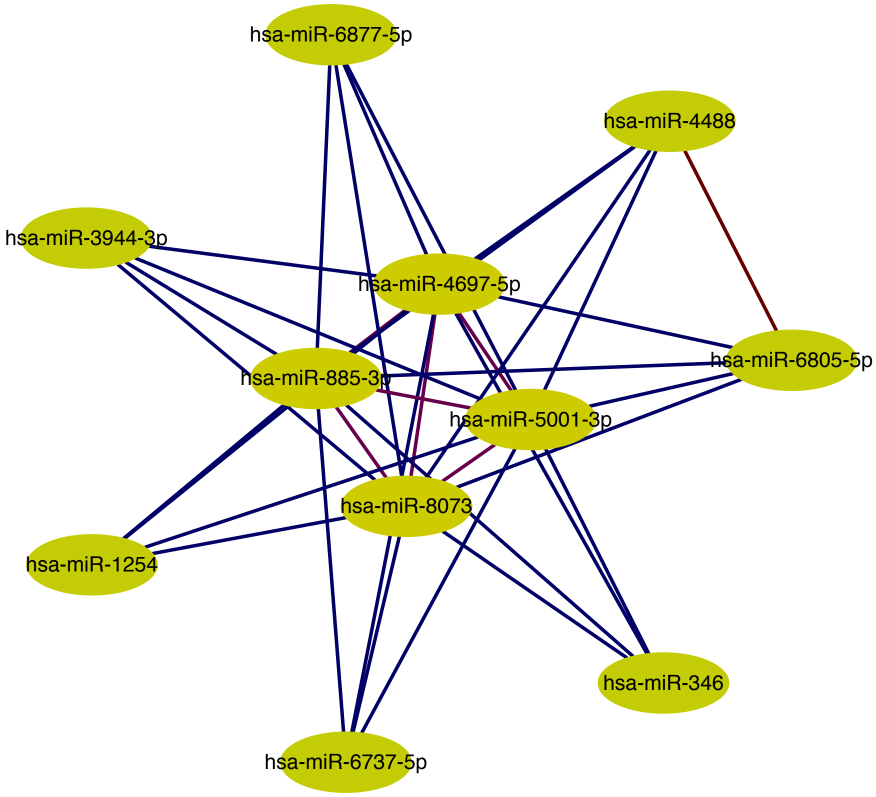
hsa-miR-6877-5p
hsa-miR-4488
hsa-miR-3944-3p
hsa-miR-4697-5p
hsa-miR-6805-5p
hsa-miR-885-3p
hsa-miR-5001-3p
hsa-miR-8073
hsa-miR-1254
hsa-miR-346
hsa-miR-6737-5p

Module 3

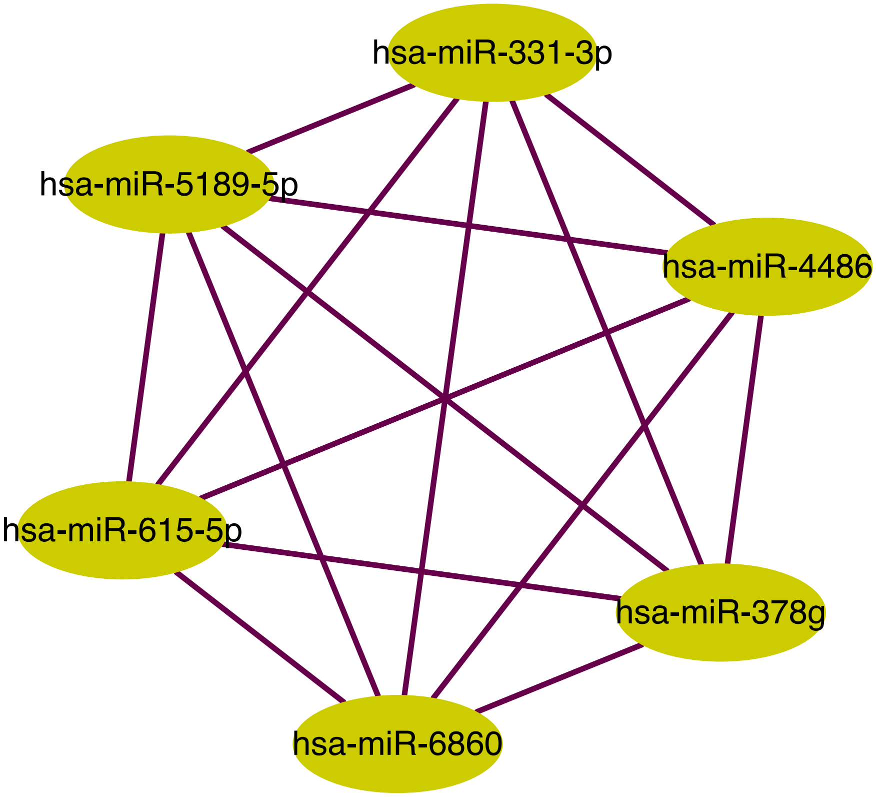
hsa-miR-331-3p
hsa-miR-5189-5p
hsa-miR-4486
hsa-miR-615-5p
hsa-miR-378g
hsa-miR-6860

Module 4

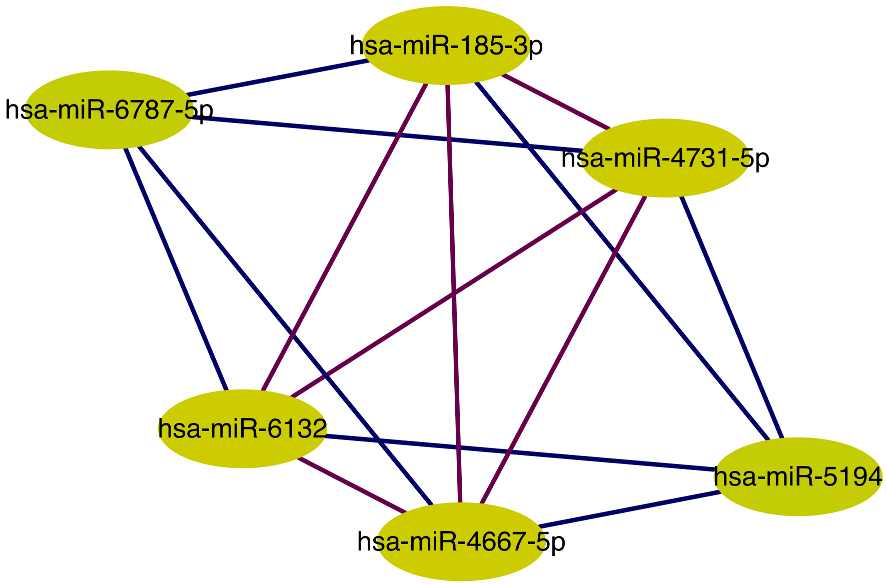
hsa-miR-185-3p
hsa-miR-6787-5p
hsa-miR-4731-5p
hsa-miR-6132
hsa-miR-5194
hsa-miR-4667-5p

Module 5

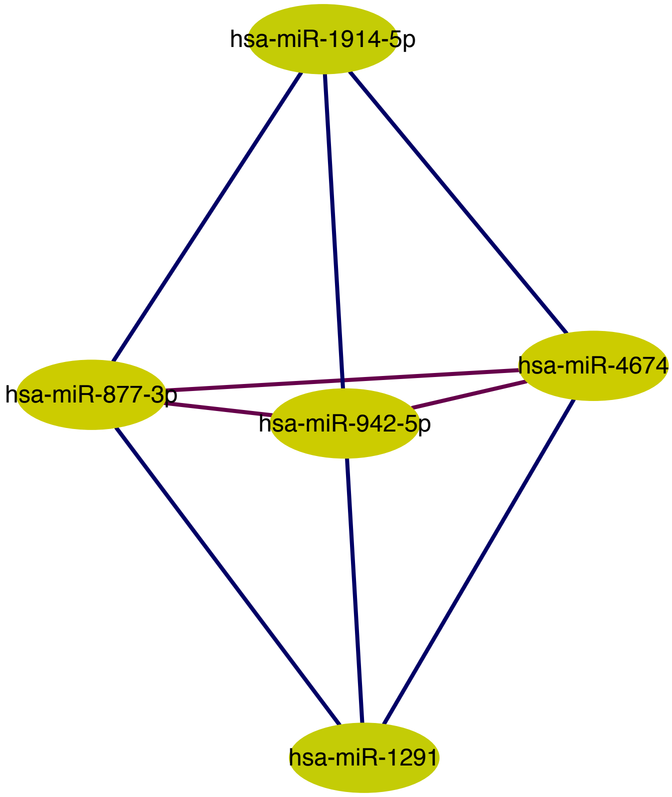
hsa-miR-1914-5p
hsa-miR-877-3p
hsa-miR-4674
hsa-miR-942-5p
hsa-miR-1291

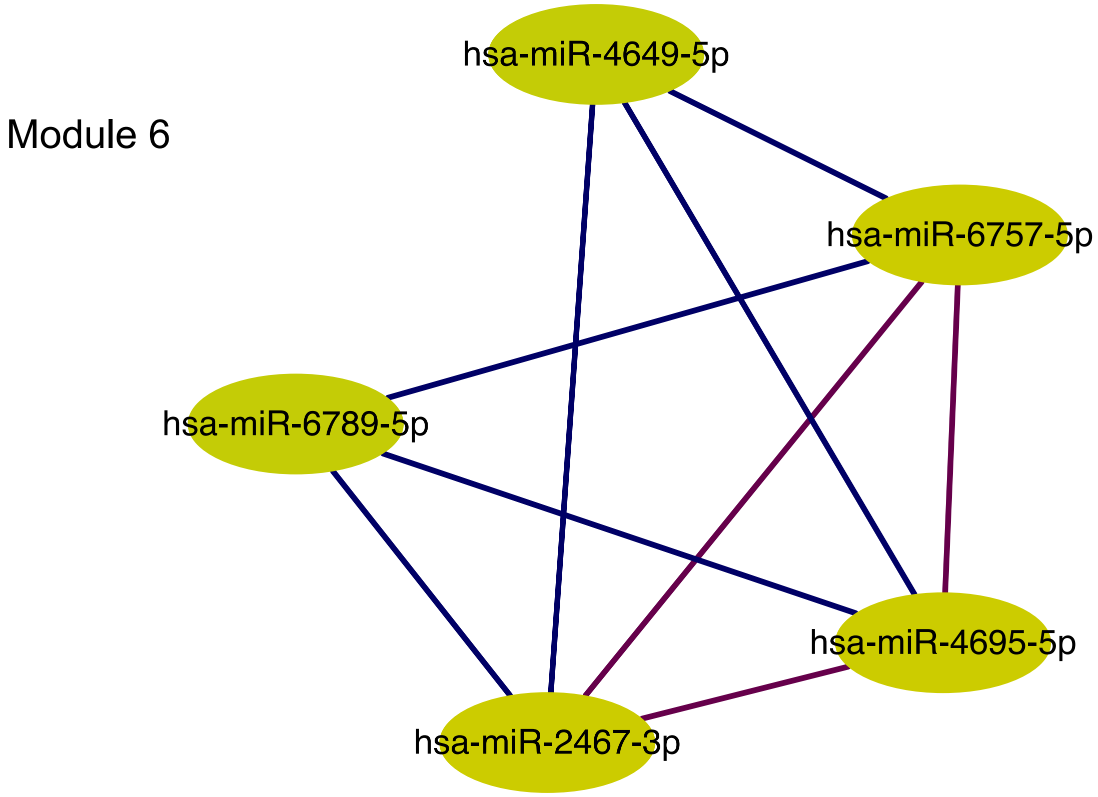
Module 6
hsa-miR-4649-5p
hsa-miR-6757-5p
hsa-miR-6789-5p
hsa-miR-4695-5p
hsa-miR-2467-3p

Module 7

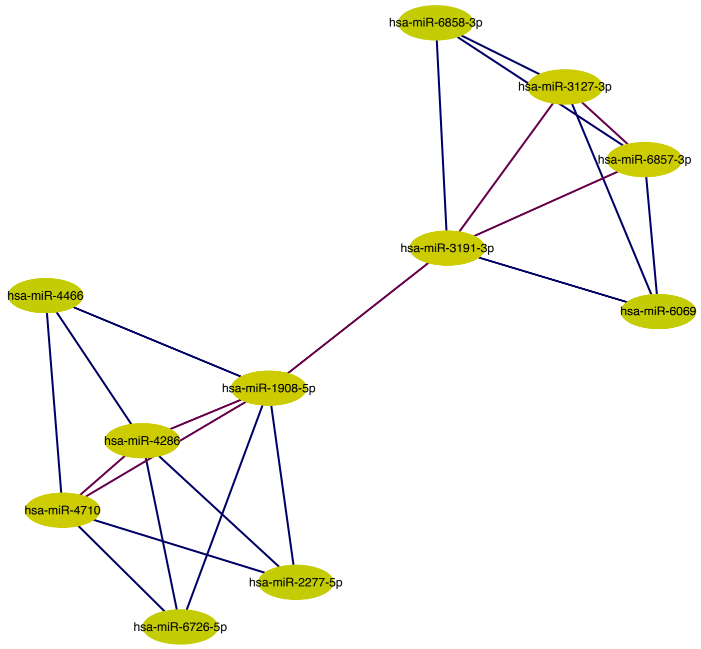
hsa-miR-6858-3p
hsa-miR-3127-3p
hsa-miR-6857-3p
hsa-miR-3191-3p
hsa-miR-6069
hsa-miR-4466
hsa-miR-1908-5p
hsa-miR-4286
hsa-miR-4710
hsa-miR-2277-5p
hsa-miR-6726-5p

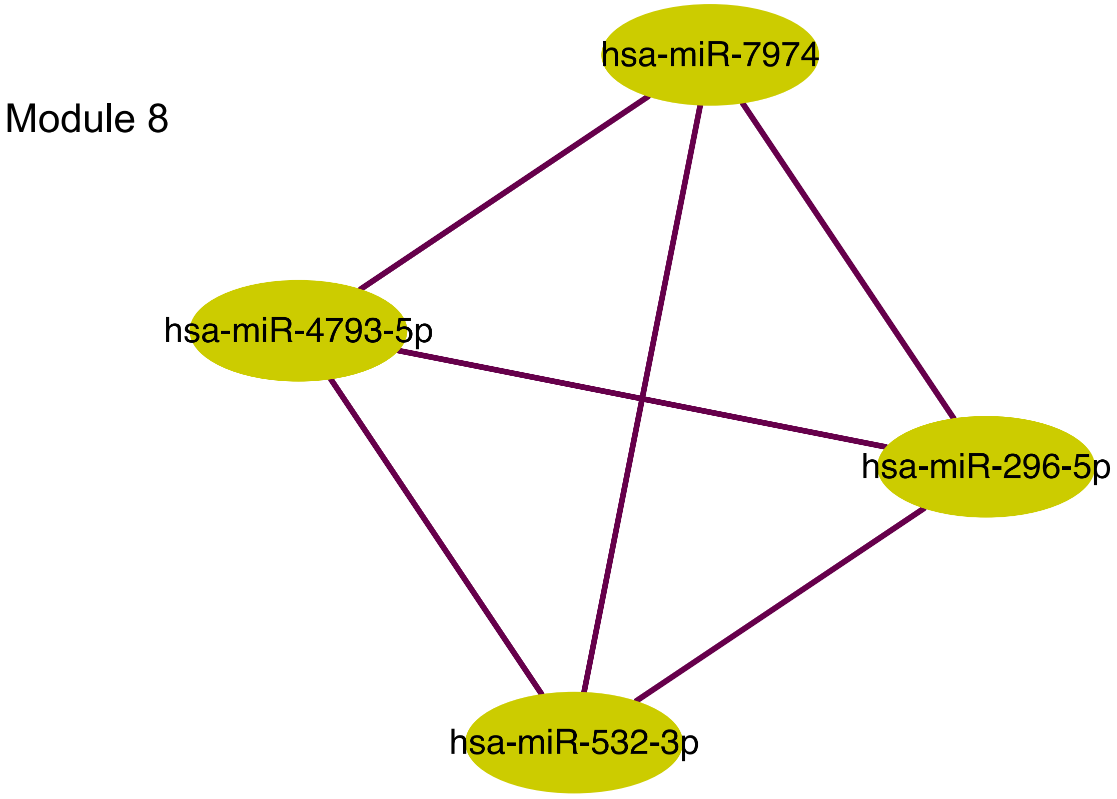
Module 8
hsa-miR-7974
hsa-miR-4793-5p
hsa-miR-296-5p
hsa-miR-532-3p

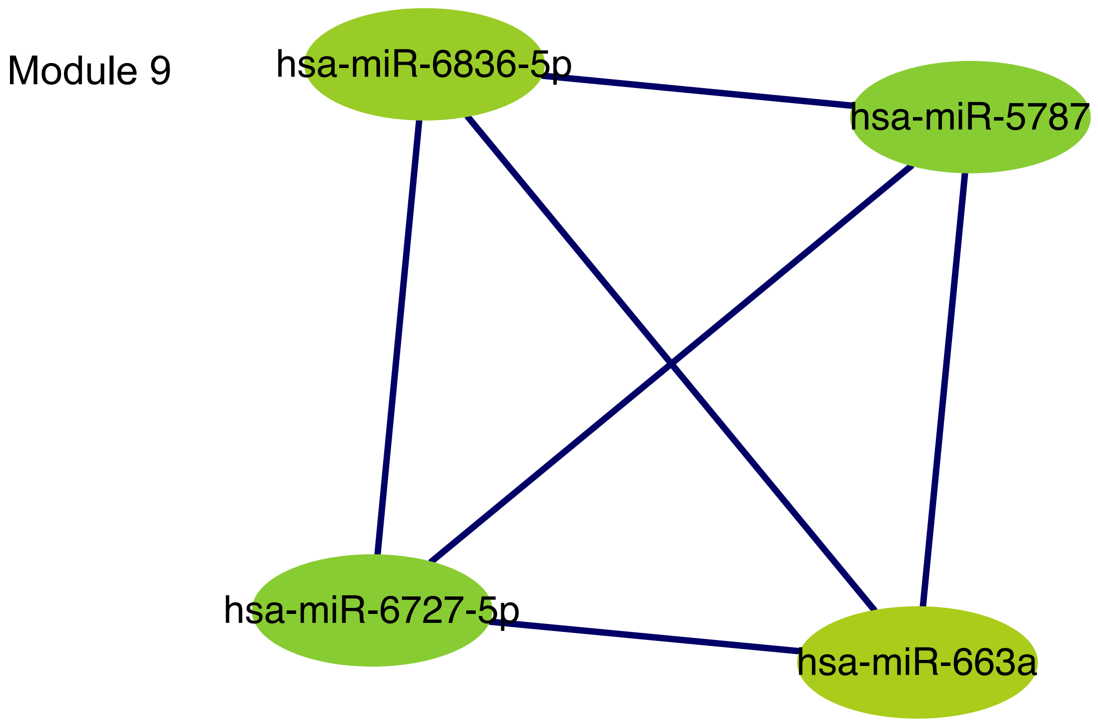
Module 9
hsa-miR-6836-5p
hsa-miR-5787
hsa-miR-6727-5p
hsa-miR-663a

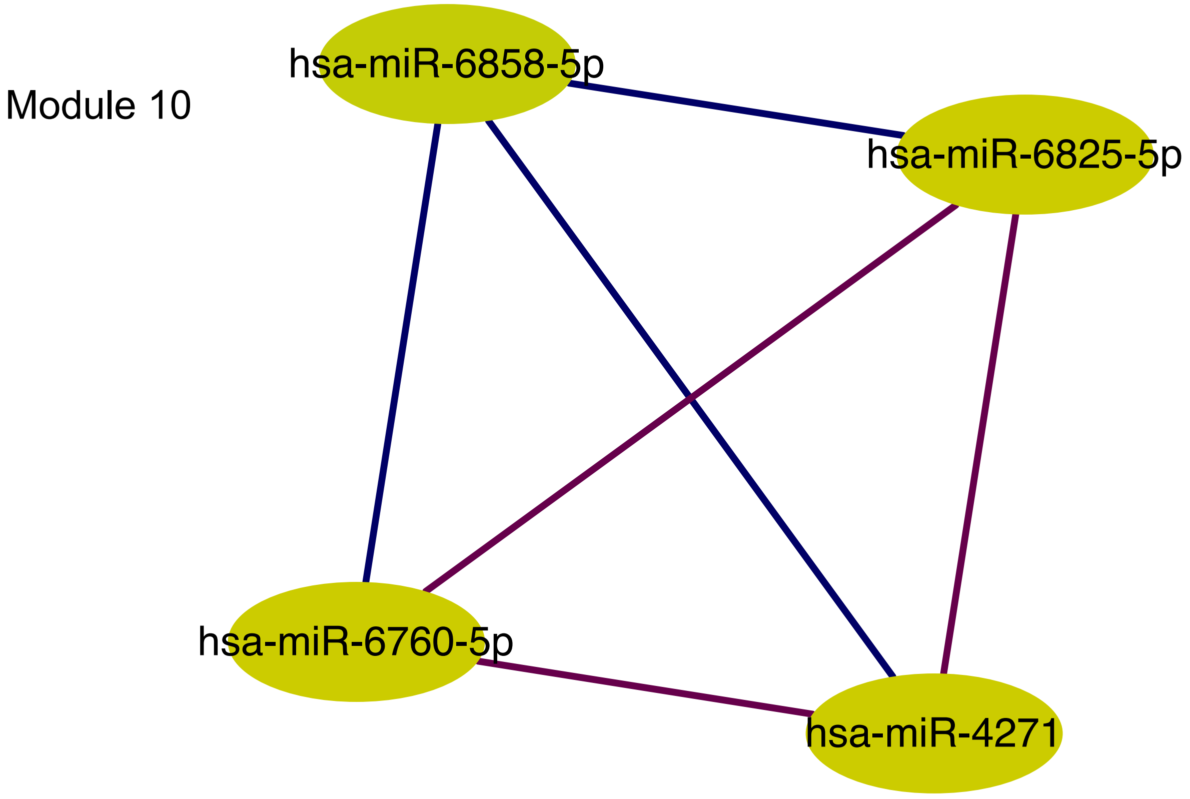
Module 10
hsa-miR-6858-5p
hsa-miR-6825-5p
hsa-miR-6760-5p
hsa-miR-4271

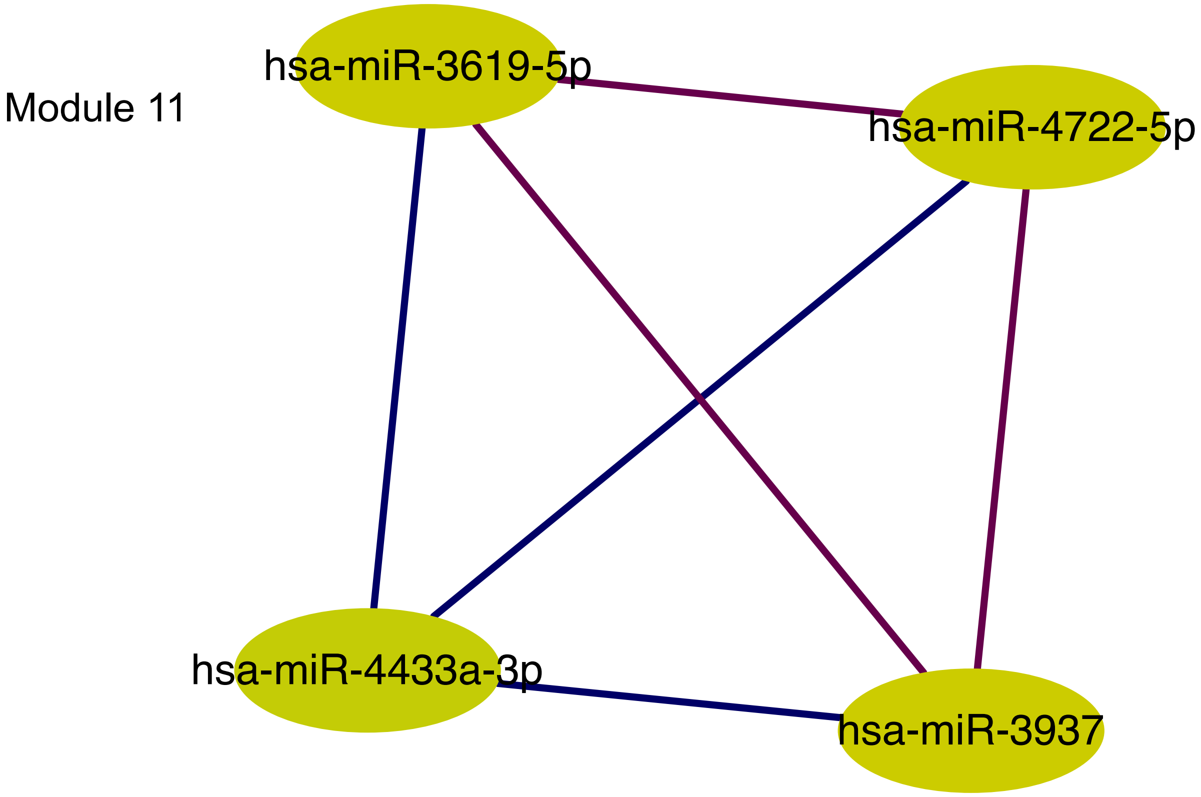
Module 11
hsa-miR-3619-5p
hsa-miR-4722-5p
hsa-miR-4433a-3p
hsa-miR-3937

Module 12

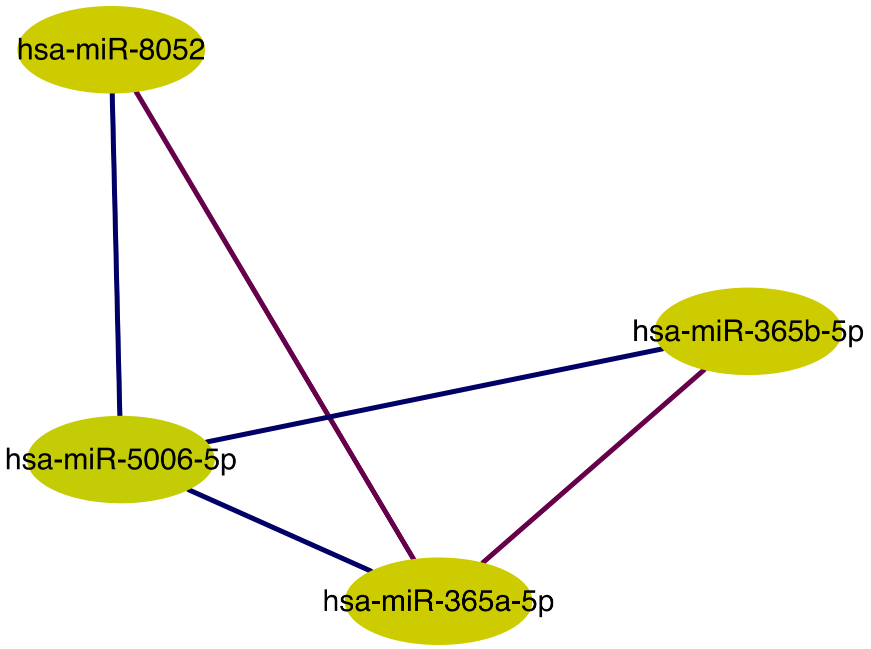

Module 13

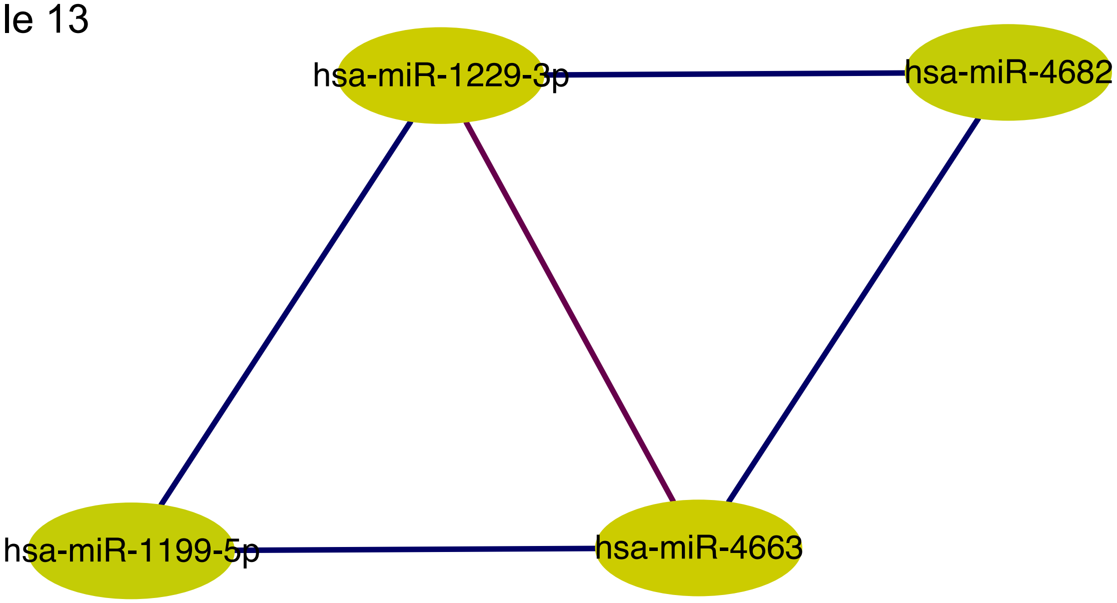

Module 14

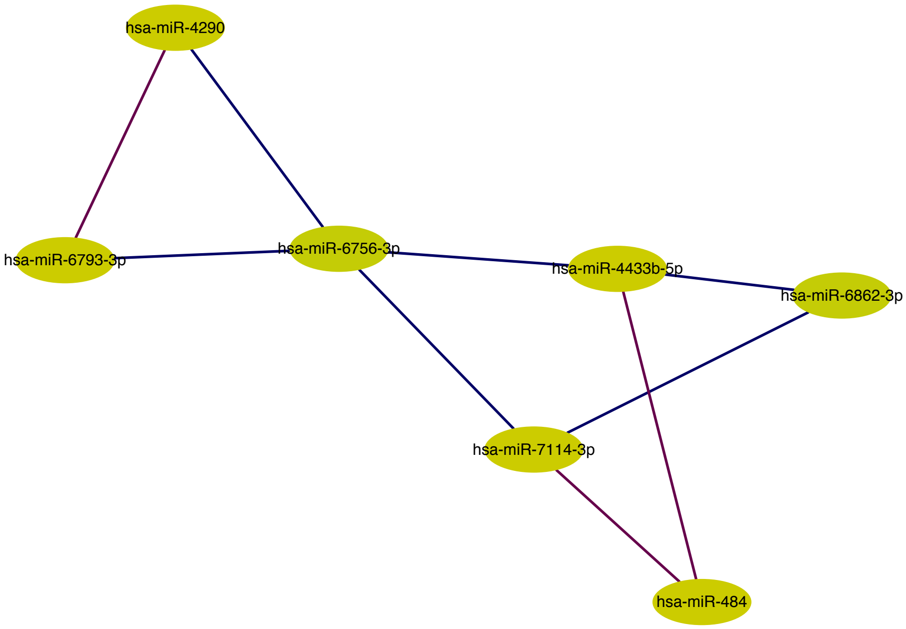
hsa-miR-4290
hsa-miR-6793-3p
hsa-miR-6756-3p
hsa-miR-4433b-5p
hsa-miR-6862-3p
hsa-miR-7114-3p
hsa-miR-484

Module 15

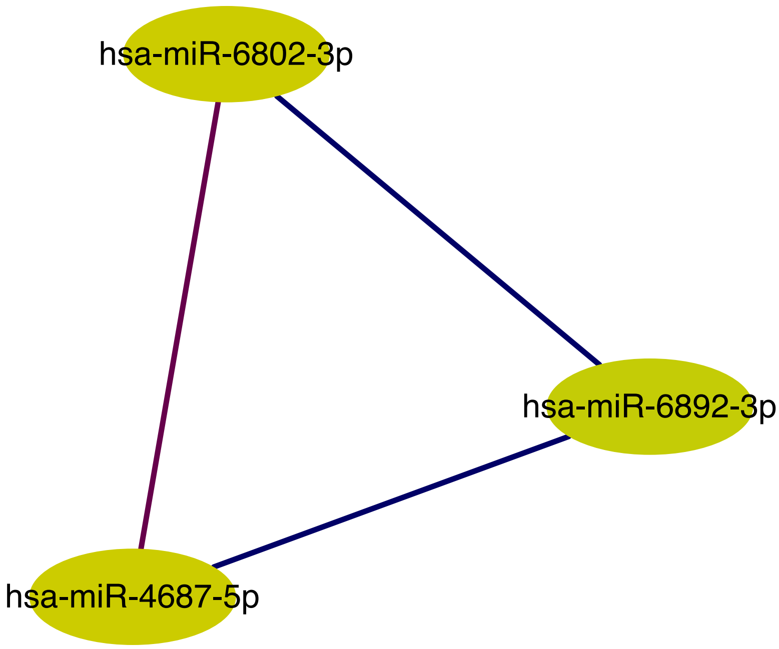

Module 16

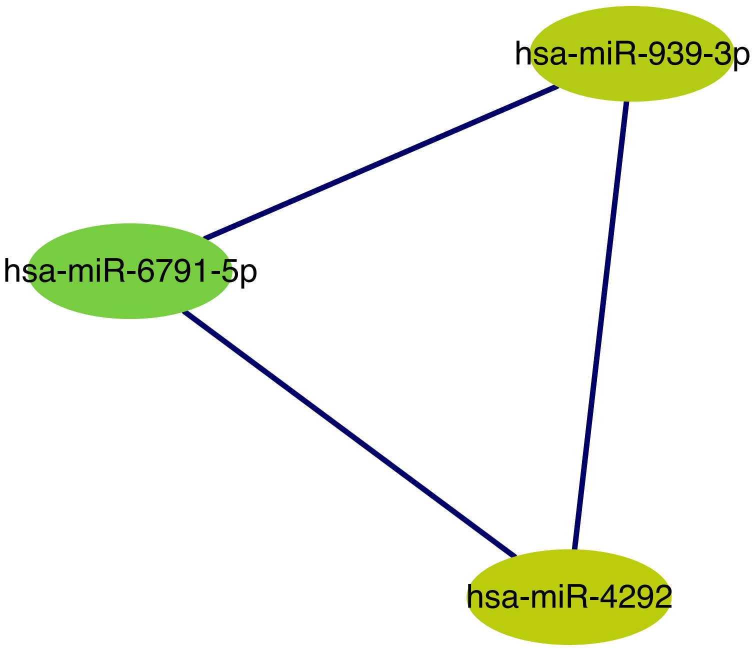

Module 17

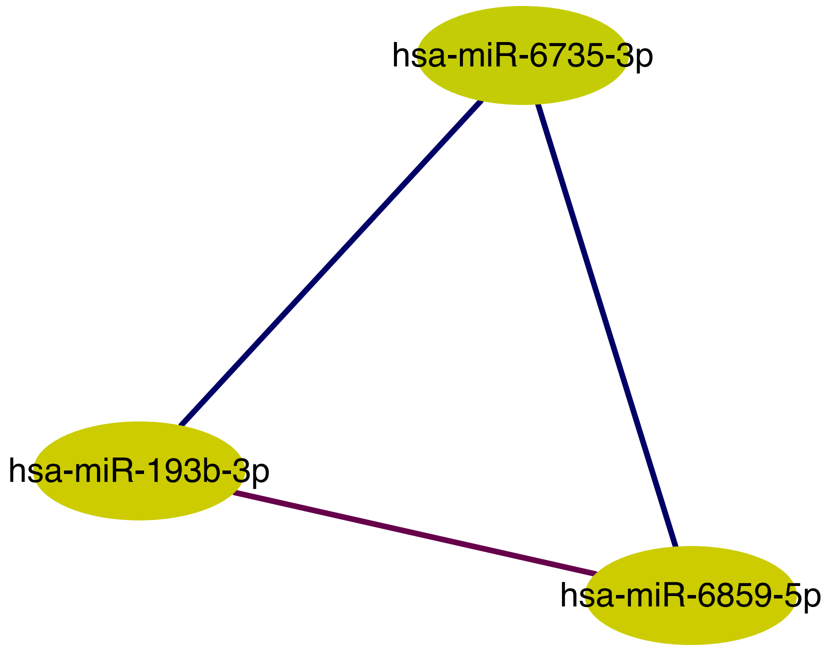

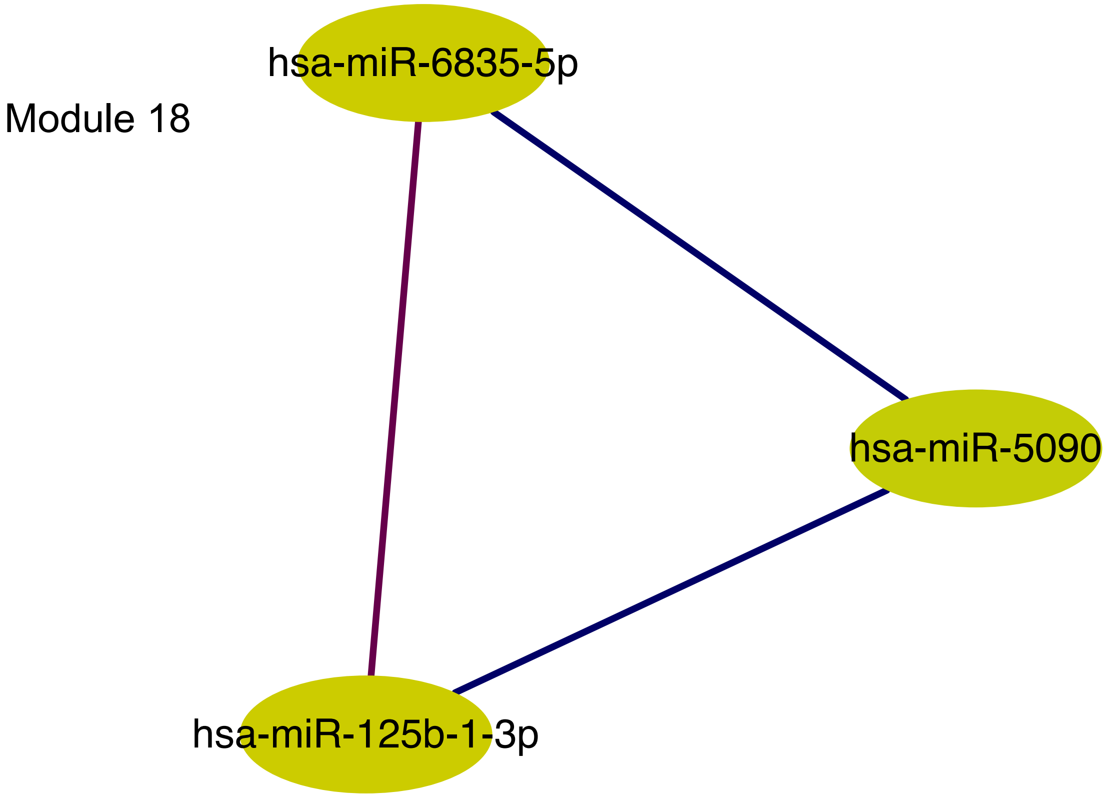
Module 18
hsa-miR-6835-5p
hsa-miR-5090
hsa-miR-125b-1-3p
