# Supplementary figures and images for: miRNA Mediated Noise Making of 3′UTR Mutations in Cancer
Source: Genes (Basel). 2018 Nov 12;9(11):545. doi: 10.3390/genes9110545 (PMC6267165; doi:10.3390/genes9110545)

## Degree of energy change

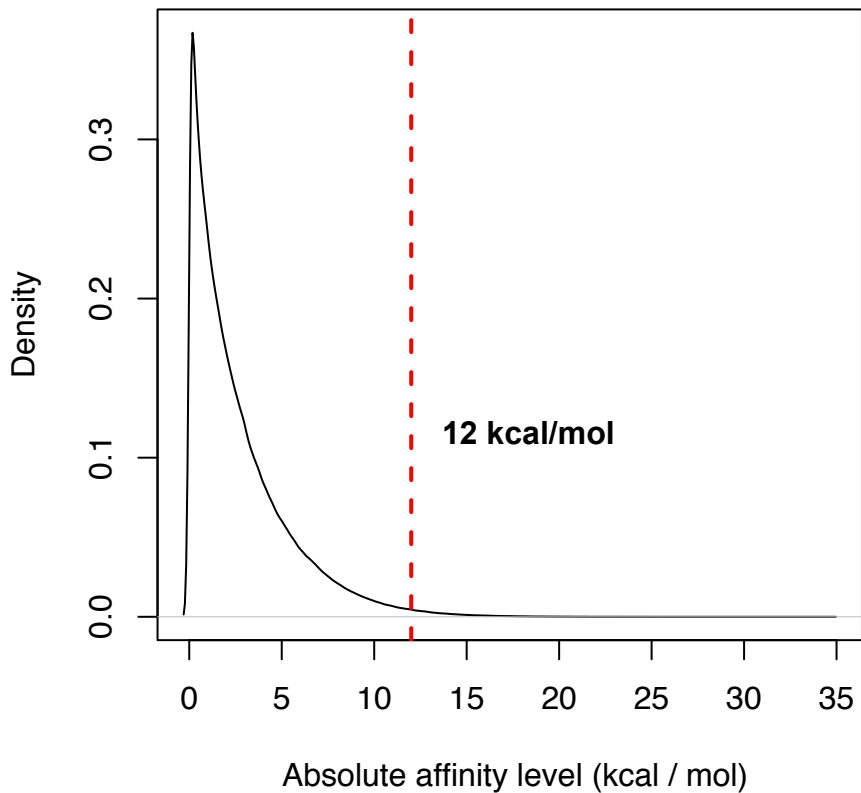

Supplement: Supplementary file 1 [file genes-09-00545-s001.zip › genes-382433-Supplementary/Supplement Figure S1.pdf]

A

Tumor

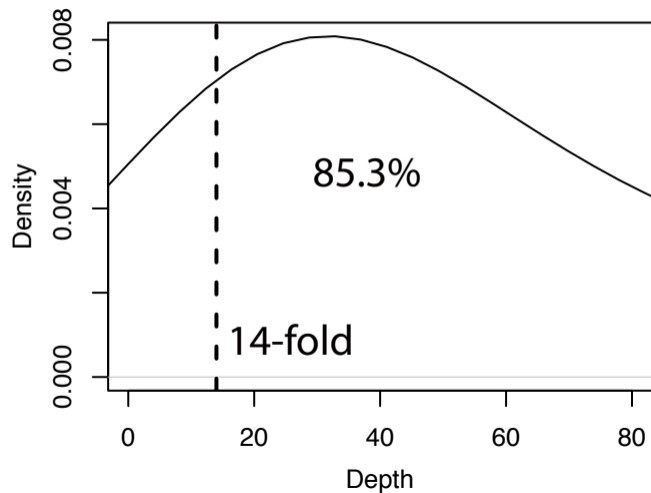

B

Normal

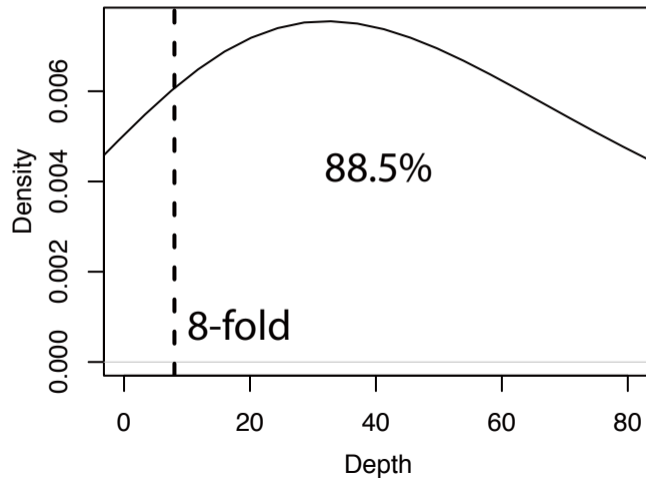

Supplement: Supplementary file 1 [file genes-09-00545-s001.zip › genes-382433-Supplementary/Supplement Figure S2.pdf]
